# The Practice of Wearing Surgical Masks during the COVID-19 Pandemic

**DOI:** 10.3201/eid2608.201498

**Published:** 2020-08

**Authors:** Cho-Han Chiang, Cho-Hung Chiang, Cho-Hsien Chiang, Yee-Chun Chen

**Affiliations:** National Taiwan University College of Medicine, Taipei, Taiwan (Cho-Han Chiang, Y.-C. Chen);; Fu-Jen Catholic University, Taipei (Cho-Hung Chiang);; Chung Shan Medical University, Taichung, Taiwan (Cho-Hsien Chiang);; National Taiwan University Hospital, Taipei (Y.-C. Chen)

**Keywords:** mask, COVID-19, coronavirus disease, SARS-CoV-2, severe acute respiratory syndrome coronavirus 2, viruses, respiratory infections, zoonoses, surgical mask, pandemic, policy, source control, community, evidence-based practice, Taiwan, Singapore

**To the Editor:** We read with interest the meta-analysis conducted by Xiao et al. (*1*) that found no significant reduction in influenza transmission with the use of surgical masks in the community, based on 10 randomized controlled trials. Nevertheless, mechanistic studies found that surgical masks could prevent transmission of human coronavirus and influenza virus infections if worn by infected persons (*2*). The authors pointed out the limitations of their study: small sample size and suboptimal adherence in the mask-wearer group (*1*). Recommendations on masks in the community vary across countries during the coronavirus disease (COVID-19) pandemic (*3*); studies have reported mixed results (*2*,*4*,*5*). 

Epidemiologic data may provide an alternative insight. As of April 3, 2020, Taiwan recorded 348 COVID-19 cases (1.46/100,000 population), of which 48 (13.8%) were local cases. Singapore recorded 1,114 cases (19.07/100,000 population), of which 572 (51.3%) were local cases. Taiwan and Singapore both employed stringent measures. Taiwan recommended the use of masks early in the pandemic. In contrast, Singapore did not recommend the use of masks until April 3 and initiated its Stay Home policy on April 7.

Before the 2003 severe acute respiratory syndrome coronavirus epidemic in Taiwan, only persons with open tuberculosis wore masks in public. During the epidemic, wearing a mask in public was stigmatized. Thereafter, we educated the public to wear masks as a practice of respiratory hygiene. Although evidence is limited for their effectiveness in preventing transmission of severe acute respiratory syndrome coronavirus 2, either for source control or to reduce exposure, the wearing of masks by healthy persons may prevent potential asymptomatic or presymptomatic transmission (*3*). This marginal reduction in transmission may produce substantial results, particularly when it is implemented early. Taiwan had the foresight to create a large stockpile of medical and surgical masks; other countries or regions might now consider doing so as part of future pandemic plans (*3*).
